# Omega-3 fatty acids status in human subjects estimated using a food frequency questionnaire and plasma phospholipids levels

**DOI:** 10.1186/1475-2891-11-46

**Published:** 2012-07-09

**Authors:** Véronique Garneau, Iwona Rudkowska, Ann-Marie Paradis, Gaston Godin, Pierre Julien, Louis Pérusse, Marie-Claude Vohl

**Affiliations:** 1 Department of Food Science and Nutrition, Laval University, Québec, Canada; 2 Genomics and Endocrinology, Laval University Medical Center, Québec, Canada; 3 Institute of Nutraceuticals and Functional Foods, Québec, Canada; 4 Faculty of Nursing, Laval University, Québec, Canada; 5 Department of Social and Preventive Medicine, Laval University, Québec, Canada

**Keywords:** Food frequency questionnaire, Plasma phospholipids, *n*-3 PUFA, Biomarker, Gas chromatography

## Abstract

**Background:**

Intakes of omega-3 (*n*-3) fatty acids (FA) are associated with several health benefits. The aim of this study was to verify whether intakes of *n*-3 FA estimated from a food frequency questionnaire (FFQ) correlate with *n*-3 FA levels measured in plasma phospholipids (PL).

**Methods:**

The study sample consisted of 200 French-Canadians men and women aged between 18 to 55 years. Dietary data were collected using a validated FFQ. Fasting blood samples were collected and the plasma PL FA profile was measured by gas chromatography.

**Results:**

Low intakes of *n*-3 long-chain FA together with low percentages of *n*-3 long-chain FA in plasma PL were found in French-Canadian population. Daily intakes of eicosapentaenoic acid (EPA), docosapentaenoic acid (DPA) and docosahexaenoic acid (DHA) were similar between men and women. Yet, alpha-linolenic acid (ALA) and total *n*-3 FA intakes were significantly higher in men compared to women (ALA: 2.28 g and 1.69 g, p < 0.0001, total *n*-3 FA: 2.57 g and 1.99 g, p < 0.0001; respectively). In plasma PL, DPA and DHA percentages were significantly different between men and women (DPA: 1.03% and 0.88%, p < 0.0001, DHA: 3.00% and 3.43%, p = 0.0005; respectively). Moreover, DHA (men: r = 0.52, p < 0.0001; women: r = 0.57, p < 0.0001) and total *n*-3 FA (men: r = 0.47, p < 0.0001; women: r = 0.52, p < 0.0001) intakes were positively correlated to their respective plasma PL FA levels. In women, EPA (r = 0.44, p < 0.0001) and DPA (r = 0.23, p = 0.02) intakes were also correlated respectively with EPA and DPA plasma PL FA percentages.

**Conclusion:**

Estimated *n*-3 long-chain FA intake among this young and well-educated French-Canadian population is lower than the recommendations. Further, FFQ data is comparable to plasma PL results to estimate DHA and total *n*-3 FA status in healthy individuals as well as to evaluate the EPA and DPA status in women. Overall, this FFQ could be used as a simple, low-cost tool in future studies to rank *n*-3 FA status of individuals.

## Introduction

Intakes of omega-3 (*n*-3) fatty acids (FA) are associated with numerous health benefits [1]. Particularly, *n*-3 polyunsaturated fatty acids (PUFA) help to modulate factors contributing to the metabolic syndrome characterised by abdominal obesity, insulin resistance, dyslipidemia and hypertension [2-4]. *n*-3 PUFA are also known to have beneficial effects on cardiovascular disease risk [5-8] and favourable effects on symptoms of depression [9], weight status [10], postprandial satiety [11], insulin resistance [12] and inflammatory diseases [13]. Thus, there is a need to have an accurate and non-invasive tool to estimate *n*-3 PUFA intakes and enable analyses on health in large epidemiological studies.

Numerous food frequency questionnaires (FFQ) have previously been developed to estimate specifically FA intake. In the literature, validation studies for *n*-3 FA specific FFQ were conducted against other reported dietary questionnaires, biomarkers or other methods [14,15]. The current FFQ was previously validated with a three-day food record that demonstrated significant correlation coefficients for eicosapentaenoic acid (20:5; EPA) (r = 0.33, p ≤ 0.01) and docosahexaenoic acid (22:6; DHA) (r = 0.30, p ≤ 0.01) in French-Canadian women [16]. Correlations between the FFQ and the three-day food record have not been examined for other FA, such as alpha-linolenic acid (18:3; ALA) and docosapentaenoic acid (22:5; DPA).

Plasma *n*-3 FA are considered good biomarkers to evaluate the validity of dietary recall methods of *n*-3 FA intake since they are largely derived from exogenous sources [17]. However, tissue *n*-3 FA levels are subjected to individual variations due to digestion, absorption, metabolism, use of the FA as energy, genetic factors, sex hormones, etc. [14,18-20]. In the same manner, dietary recall methods may inherit inaccuracies due to underreporting of dietary fat intake or errors associated with databases [17,19,21]. Thus, the use of biomarkers may be a good method to validate an FFQ. Overall, the objective of this study was to verify whether *n*-3 dietary intakes estimated by a validated FFQ correlate with *n*-3 FA levels measured in plasma phospholipids (PL) in a healthy French-Canadian population.

## Methods

### Study population

200 subjects, including 100 men and 100 women, aged between 18 and 55 years were randomly selected from a previous study conducted in two phases from May 2004 to December 2004 and from March 2006 to April 2007 in the Quebec City metropolitan area [22]. Briefly, advertisements in local newspapers and radio stations, in addition to electronic group messages sent to university and hospital employees, were used to recruit participants. The following data was collected from each of the study participants during the visit: lifestyle habits and socio-demographic questionnaires, anthropometric measures, fasting blood samples, and the FFQ. All participants signed an informed consent form to participate to this study, which has been approved by the Ethics Committee of Laval University.

### Dietary intake

A FFQ was used to assess dietary intakes of Quebecers. This FFQ has previously been validated and evaluated for reproducibility in the French-Canadian population [16,23]. The FFQ had 91 items. The nutrient content of each item on the FFQ was selected from the Nutrition Data System (NDS) for Research (Nutrition Coordination Center, University of Minnesota, Minneapolis, MN) in 2001. Specifically, the nutrients values of the food items from NDS were chosen based on the nutrient values which represent foods available in the Quebec market. In addition, these nutrient values were compared to the Canadian databases for accuracy. Frequency of food consumed was determined by asking how often a participant consumed each item per day, week, month or none at all during the previous month. To obtain a better estimation of the portion consumed by the participant, examples of portion size were provided. Information on *n*-3 supplement intake was also collected and included in total *n*-3 intake estimates.

### Fatty acid composition of plasma phospholipids

Fasting blood samples were collected into vacutainer tubes containing EDTA. After centrifugation, plasma was aliquoted and stored. Samples were stored at −80 °C for 2 to 5 years*.* Erythrocytes are considered the gold standard to measure the long-term *n*-3 status. Yet, numerous studies have reported strong correlations between FA content of erythrocytes and plasma PL; therefore, plasma PL may be considered as a valid biomarker to reflect n-3 status over the last month [21,24]. Extraction of plasma lipids was made using a chloroform-methanol mixture (2:1, vol/vol). Total PL were separated by thin layer chromatography using a combination of isopropyl ether and acetic acid and fatty acids of isolated PL were then methylated. Capillary gas chromatography was then used to obtain FA profiles. The technique used for plasma analyses has been previously validated [25]. Values of FA levels are expressed as percent of total FA in plasma PL.

### Statistical analysis

Means (±SDs) were calculated by sex for dietary intakes, plasma PL *n*-3 FA percentages and other subjects’ characteristics. Relationships between *n*-3 PUFA dietary intakes and plasma PL *n*-3 FA levels were assessed by Spearman’s correlation coefficients. Correlations were computed for men and women separately and adjusted for age and energy intake. With a sample size of 100 individuals in each group calculated with 80% power and *P* ≤ 0.05 (two-sided), we can expect to find a correlation coefficient of 0.26 or stronger [26]. In addition, participants were classified into quartiles according to FFQ- dietary intakes and plasma PL *n*-3 FA levels. The accuracy of the FFQ to classifying individuals by gender into same or adjacent plasma-determined quartiles was determined. All analyses were performed with SAS, version 9.1 (SAS Institute Inc., Cary, North Carolina). *P*-values ≤0.05 were considered statistically significant.

## Results

Analyses were performed on 200 participants (100 men and 100 women). Subjects’ characteristics are presented in Table 1. The sample consisted of a young adult population with a high percentage of study participants having a college or university degree. Further, there was no significant difference between men and women for these presented subjects’ characteristics. A personal income over 50,000$ (CDN)/year was more common in men than in women. Ten percent of participants were considered as current smokers.

**Table 1:** Subject’s characteristics (n = 200)

Women, %	50	
Age (years)	34.3	10.2
BMI (Kg/m²)	29.1	6.2
Education		
high school, %	9.5	
college degree, %	32.5	
university degree, %	58.0	
Personal income, $ *		
< 12,000, %	27.6	
12,000 – 29,999, %	25.6	
30,000 – 49,999, %	26.8	
≥ 50,000, %	20.0	
Current smokers, % *	9.6	

Men had a significantly greater energy intake than women and a higher proportion of energy intake coming from fat. Women, on the other hand, had a higher proportion of energy intake derived from carbohydrates. As presented in Table 2, EPA, DPA and DHA daily intakes were comparable for men and women. However, ALA and total *n*-3 FA intakes were significantly higher in men than in women. From the FFQ data, ALA sources were mainly from higher-fat processed foods and canola oil. The main sources of DPA included meat and fish products. The main contributors of EPA and DHA were the fried fish and seafood as well as fish, molluscs and crustaceans. Further, approximately 12.1% of subjects reported taking *n*-3 capsules as a dietary supplement.

**Table 2:** Daily dietary intakes

	**Men (n = 100)**		**Women (n = 100)**		
	**Mean**	**SD**	**Mean**	**SD**	***p***
Energy, kcal	2810	854	2140	533	<0.0001
Carbohydrates, % of energy	47.3	6.2	49.8	6.5	0.005
Proteins, % of energy	17.0	2.5	16.7	2.2	0.34
Fat, % of energy	34.3	5.1	32.8	5.5	0.05
Saturated fat, % of energy	11.9	2.6	11.3	2.6	0.15
MUFA, % of energy	14.1	2.5	13.4	2.7	0.06
PUFA, % of energy	5.6	1.3	5.5	1.4	0.51
ALA, g	2.28	1.13	1.69	0.76	<0.0001
EPA, g	0.08	0.08	0.09	0.09	0.28
DPA, g	0.04	0.02	0.03	0.02	0.29
DHA, g	0.17	0.14	0.18	0.15	0.70
total n-3, g	2.57	1.14	1.99	0.85	<0.0001

total n-3 : ALA + EPA + DPA + DHA.

Mean plasma PL FA are shown in Table 3. Significant sex differences were observed for plasma PL DPA and DHA. Women presented the highest percentage of plasma PL DHA and men had greater plasma PL DPA percentage. In men and women, plasma PL DHA percentage was the FA found in the greatest percentage whereas ALA was found in the lowest percentage. In addition, women tended to have a higher percentage of total *n*-3 FA in plasma PL than men.

**Table 3:** Participants plasma PL FA concentrations

	**Men (n = 100)**		**Women (n = 98)**		
	**Mean**	**SD**	**Mean**	**SD**	***p***
ALA, %	0.15	0.14	0.19	0.14	0.06
EPA, % *	1.09	0.56	1.08	0.61	0.82
DPA, %	1.03	0.16	0.88	0.24	<0.0001
DHA, %	3.00	0.80	3.43	0.92	0.0005
total n-3, %	5.33	1.19	5.73	1.67	0.08

* for women n = 98.

Correlations between dietary intakes of *n*-3 FA and the levels of the same FA in plasma are presented in Table 4. Adjustment for *n*-3 supplementation did not alter the correlation coefficients (data not shown). In general, significant and positive relationships were observed for DHA and total *n*-3 FA in both genders. In women, significant and positive relationships were also observed for EPA and for DPA. On the other hand, no relationships were found for ALA in either gender.

**Table 4:** Spearman correlation coefficients between plasma PL and FFQ FA *

	**Men (n = 100)**		**Women (n = 100)**	
	**r**	***p***	**r**	***p***
ALA	0.14	0.18	0.19	0.06
EPA **	0.11	0.26	0.44	<0.0001
DPA	−0.08	0.46	0.23	0.02
DHA	0.52	<0.0001	0.57	<0.0001
total n-3	0.47	<0.0001	0.52	<0.0001

* Adjusted for age and energy intake.

** for women n = 98.

Further, the accuracy of the FFQ to rank individuals into the same or adjacent plasma quartiles was 79% and 77% for total n-3, 85% and 78% for DHA, 57% and 68% for DPA, 60% and 75% for EPA, and 72% and 66% for ALA for men and women, respectively.

## Discussion

Results from this study demonstrate that the FFQ appears to provide adequate information on *n*-3 intake in relation to corresponding FA levels in plasma PL. In particular, dietary intakes estimated by a FFQ were correlated with percentages of DHA and total *n*-3 FA in plasma PL.

FFQ estimates of dietary intakes for EPA, DPA and DHA in this young and well-educated French-Canadian population were comparable to results reported in previous studies [27-33]. Yet, ALA and total *n*-3 FA intakes were higher compared to intakes observed in earlier studies [27-33]. The estimated daily dietary intake of ALA in most European countries and United States is of 1.3 to 1.7 g [34,35]. Thus, the higher intake of ALA may be due to the type of vegetable oil used by this population and thus may have beneficial effects on the cardiovascular disease risk profile. Yet, the consumption of EPA and DHA in this young and well-educated French-Canadian study population was below the level of 0.5 g/day recommended to decrease the risk of cardiovascular disease [36,37]. The combined daily intake of EPA and DHA was 0.25 g for men and 0.27 g for women. Similarly, Lucas et al., examined the *n*-3 FA status of individuals living in the Quebec City metropolitan area and established that the mean daily intake of EPA and DHA was of 0.29 g [38]. However, in our study the *n*-3 FA intake was higher than that reported in the NHANES 1999–2000, where mean intake of EPA plus DHA was approximately 0.1 g/d [39]. Nevertheless, the present intake of EPA and DHA is much lower than the 1.6 g daily intake observed in the Japanese population, which has one of the lowest rates of heart disease in the world [40].

Even though the FFQ estimates are comparable to other studies, percentages of *n*-3 FA in plasma PL of the participants were lower than the percentages previously reported in the literature [17,30,41]. In particular, plasma DHA and total *n*-3 percentage were much lower compared to the average from the Melbourne Collaborative Cohort Study (MCSS) (4.0-4.2% DHA and 6.6-6.8% total *n*-3) [30], the European Prospective Investigation into Cancer (EPIC) -Norfolk United Kingdom cohort (5.13-5.28% DHA and 7.81-7.96% total n-3 FA) [41], and the entire EPIC cohort (4.6-6.6% DHA, and 6.7-10.4% total *n*-3)[17]. According to several studies, FA composition stay stable for many years when plasma is stored at −80 °C [19,21,42]; therefore, the poor levels of DHA and total *n*-3 FA observed in the current study are unlikely to be explained by FA oxidation. Overall, results indicate lower levels of *n*-3 FA in plasma PL in the French-Canadian population.

Results show a modest relationship between FFQ estimates of dietary intakes and percentages of *n*-3 FA in plasma PL, except for ALA in women and ALA, EPA and DPA in men. The results of the present study are in agreement with results reported in previous studies [15,27,28,32,43,44]. First, a validity study was conducted in Australia for a non-specific FFQ using plasma PL [32]. The results demonstrate significant relationships only for DHA (0.32) and total long-chain *n*-3 (0.38) [32]. Secondly, Hodge et al. [27], obtained similar results for ALA, EPA, DHA and total *n*-3 FA with a general FFQ (0.07, 0.18, 0.40 and 0.31, respectively). Thirdly, Arsenault et al. [28] demonstrated correlation coefficients of 0.37 for EPA, 0.48 for DHA and 0.48 for total *n*-3 FA between an *n*-3 PUFA specific FFQ and plasma PL among elderly with cognitive impairment. Finally, earlier studies conducted with other biomarkers, such as red blood cells, total plasma and adipose tissue, demonstrated comparable correlation coefficients [15,43-50]. Further, these results suggest that the lack of correlation between FFQ and plasma PL for ALA may be due to the fact that ALA is readily metabolized to long-chain metabolites, such as EPA and DHA [47,51] . Overall, the FFQ adequately estimated DHA and total *n*-3 FA in the population as well as EPA and DPA in women.

Taken together, results demonstrate modest correlations (0.23-0.57) between *n*-3 dietary intakes estimated using a FFQ and plasma PL. It is well accepted that perfect correlations between FA in tissues and intakes measured by FFQs are unrealistic. First, numerous factors can influence the FA composition such as the tissue used for FA profile as well as the individuals’ FA metabolism. Since the FFQ assesses the dietary intakes of the last month, plasma PL should be a reasonable biomarker to evaluate this information against as it reflects fatty acid intake over the last 21 d [27,52]. Erythrocytes are considered the gold standard to measure the long-term *n*-3 status [53]. Yet, numerous studies have reported strong correlations between FA content of erythrocytes and plasma PL; therefore, plasma PL may be considered as a reliable biomarker to reflect *n*-3 status over the last month [21,24]. Furthermore, it is well recognized that FA concentrations in tissues are subjected to other issues such as absorption, metabolism, genetic and lifestyle determinants [14,17,18,20]. Secondly, the FFQ data are influenced by the capacity of individuals to recall adequately food they consumed, portions they eat and by their knowledge of food’s composition [19,21]. In addition, the FFQ estimates are also subjected to the accuracy of the databases used [21]. In sum, the modest correlation coefficients observed in the current study support the ability of this FFQ to reflect the relative ranking of *n*-3 FA intake.

In conclusion, our results indicate intakes of approximately 0.25 to 0.27 g/d EPA and DHA in this young and educated French-Canadian population which is below the recommended 0.5 g/d EPA and DHA for cardiovascular disease risk reduction [34,36,37]. The present study also indicates that *n*-3 FA intakes examined by FFQ and validated with *n*-3 FA biomarkers, are good indicators of *n*-3 FA status. Taken as a whole, the FFQ could be used to categorize *n*-3 FA status, especially due to a lower cost compared to established biomarkers. Nevertheless, the use of the questionnaire with a less educated or older population may be less accurate compared to this particular French-Canadian population. Overall, the use of this FFQ could facilitate future studies in investigating the impact of low *n*-3 FA dietary intakes on health.

## Abbreviations

n-3, Omega-3; PUFA, Polyunsaturated fatty acid; FFQ, Food frequency questionnaire; PL, Phospholipids; FA, Fatty acids; ALA, Alphalinolenic acid; EPA, Eicosapentaenoic acid; DPA, Docosapentaenoic acid; DHA, Docosahexaenoic acid.

## Competing interests

The authors declare they have no competing interests.

## Authors’ contributions

VG performed the statistical analyses of the data and took the primary role in drafting the manuscript. IR revised and provided critical review of the manuscript. AMP, GG, PJ, LP and MCV guided the strategy of the data analyses, assisted with the interpretation of the results and provided critical review of the manuscript. GG, LP and MCV designed the INFOGENE study. All authors read and approved the final manuscript.
